# Rapid, economical diagnostic classification of ATRT molecular subgroup using NanoString nCounter platform

**DOI:** 10.1093/noajnl/vdae004

**Published:** 2024-01-16

**Authors:** Ben Ho, Anthony Arnoldo, Yvonne Zhong, Mei Lu, Jonathon Torchia, Fupan Yao, Cynthia Hawkins, Annie Huang

**Affiliations:** Division of Cell Biology, The Hospital for Sick Children, Toronto, Ontario, Canada; Arthur and Sonia Labatt Brain Tumor Research Centre, The Hospital for Sick Children, Toronto, Ontario, Canada; Department of Laboratory Medicine and Pathobiology, Faculty of Medicine, University of Toronto, Toronto, Ontario, Canada; Division of Pathology, Hospital for Sick Children, Toronto, Ontario, Canada; Division of Pathology, Hospital for Sick Children, Toronto, Ontario, Canada; Division of Cell Biology, The Hospital for Sick Children, Toronto, Ontario, Canada; Arthur and Sonia Labatt Brain Tumor Research Centre, The Hospital for Sick Children, Toronto, Ontario, Canada; Cantata Bio, LLC, Scott’s Valley, California, USA; Arthur and Sonia Labatt Brain Tumor Research Centre, The Hospital for Sick Children, Toronto, Ontario, Canada; Department of Medical Biophysics, Faculty of Medicine, University of Toronto, Toronto, Ontario, Canada; Division of Cell Biology, The Hospital for Sick Children, Toronto, Ontario, Canada; Arthur and Sonia Labatt Brain Tumor Research Centre, The Hospital for Sick Children, Toronto, Ontario, Canada; Division of Pathology, Hospital for Sick Children, Toronto, Ontario, Canada; Division of Hematology and Oncology, The Hospital for Sick Children, Toronto, Ontario, Canada; Arthur and Sonia Labatt Brain Tumor Research Centre, The Hospital for Sick Children, Toronto, Ontario, Canada; Department of Laboratory Medicine and Pathobiology, Faculty of Medicine, University of Toronto, Toronto, Ontario, Canada; Department of Medical Biophysics, Faculty of Medicine, University of Toronto, Toronto, Ontario, Canada

**Keywords:** CNS neoplasm, gene expression profiling, molecular typing, rhabdoid tumor, tumor biomarkers

## Abstract

**Background:**

Despite genomic simplicity, recent studies have reported at least 3 major atypical teratoid rhabdoid tumor (ATRT) subgroups with distinct molecular and clinical features. Reliable ATRT subgrouping in clinical settings remains challenging due to a lack of suitable biological markers, sample rarity, and the relatively high cost of conventional subgrouping methods. This study aimed to develop a reliable ATRT molecular stratification method to implement in clinical settings.

**Methods:**

We have developed an ATRT subgroup predictor assay using a custom genes panel for the NanoString nCounter System and a flexible machine learning classifier package. Seventy-one ATRT primary tumors with matching gene expression array and NanoString data were used to construct a multi-algorithms ensemble classifier. Additional validation was performed using an independent gene expression array against the independently generated dataset. We also analyzed 11 extra-cranial rhabdoid tumors with our classifier and compared our approach against DNA methylation classification to evaluate the result consistency with existing methods.

**Results:**

We have demonstrated that our novel ensemble classifier has an overall average of 93.6% accuracy in the validation dataset, and a striking 98.9% accuracy was achieved with the high-prediction score samples. Using our classifier, all analyzed extra-cranial rhabdoid tumors are classified as MYC subgroups. Compared with the DNA methylation classification, the results show high agreement, with 84.5% concordance and up to 95.8% concordance for high-confidence predictions.

**Conclusions:**

Here we present a rapid, cost-effective, and accurate ATRT subgrouping assay applicable for clinical use.

**Key Points**

- Atypical teratoid rhabdoid tumor subgrouping gene expression classifier achieved 93.6% accuracy in the test.
- Results show 84.5% concordance with DNA methylation classifier.
- Rapid and economical assay suitable for clinical needs.

**Importance of the Study**

Atypical teratoid rhabdoid tumors (ATRTs) are aggressive pediatric brain tumors with tumor heterogeneity that currently lack a standard gene expression-based molecular classification approach for clinical adoption. Here we present a novel ATRT subgrouping gene expression classifier. This assay enables rapid ATRT molecular classification and can contribute to the design of future molecularly targeted trials.

Atypical teratoid rhabdoid tumor (ATRT) is a high-grade pediatric central nervous system (CNS) tumor that is mainly found in children under the age of 2.^1^ Although it is a relatively rare disease accounting for 5% of all pediatric embryonal tumors, ATRT represents the most frequent CNS tumor that leads to mortality under the age of 6.^2^ Current diagnosis of ATRT primarily relies on histopathological examination based on its distinct morphological features. It has long been known to have a relatively simple genomic makeup with the main association with the genetic modification of *SMARCB1*. The recent edition of The World Health Organisation Classification of Tumors of the CNS tumors (WHO CNS5)^3^ recognizes 3 major ATRT subgroups ATRT-SHH (SHH), ATRT-TYR (TYR), and ATRT-MYC (MYC) with varying demographics^4,5^ Click or tap here to enter text. molecular features,^5,6^ clinical profiles^,1,4,7^ and potential therapeutic vulnerabilities.^8,9^ Although we now appreciate that ATRT is an epigenetic cancer with intertumoral heterogeneity, molecular subgrouping insights in general have not been incorporated into standard clinical diagnostic and therapeutic approaches. This highlights the importance of establishing a fast and affordable ATRT subgrouping solution applicable in clinical settings.

Currently, the molecular subgroup classification of ATRT is based on the distinct clinicopathological and molecular features of the subgroups identified by their genomic-wide transcriptomic and DNA methylomic profiles.^3–5,7,8^ While commonly used for subgrouping in scientific research, the DNA methylation or transcriptomic array-based classification methods are not ideal for clinical practice as these processes typically involve large amounts of high-quality samples, lengthy sample preparation, and high-operation costs.

A recent study has tried to circumvent these limitations by utilizing custom gene panels for tumor clustering.^9^ However, researchers are often hindered by a lack of well-verified biomarkers. In addition, due to the unsupervised nature, subgroup results may change depending on the dataset composition. These limitations make the above-mentioned methods difficult for clinical adaptation to gain reliable and accurate stratification insights.

To date, there have been multiple gene-expression-based disease subgroup predictors using a highly automated NanoString platform in clinical settings.^10^ However, its application for ATRT subgrouping has not been evaluated.

In this study, we have developed an ATRT molecular subgrouping assay and have constructed a novel ATRT classifier that we named “AClass” for research and clinical adaptation. The AClass workflow uses a minimal amount of RNA input (100 ng) to stratify ATRT tumors into molecular subgroups, based on an ATRT-specific gene signature panel derived from a minimal set of genes. Altogether, we have presented a validated novel subgroup probe panel using the NanoString platform and classification workflow that can be used for ATRT stratification from FFPE-isolated RNA samples. The feasibility and utility of the NanoString method in comparison with the DNA methylation array-based method were also examined.

## Materials and Methods

### Custom NanoString Panel Design and Construction of Ensemble Classifier

**Figure 1.**
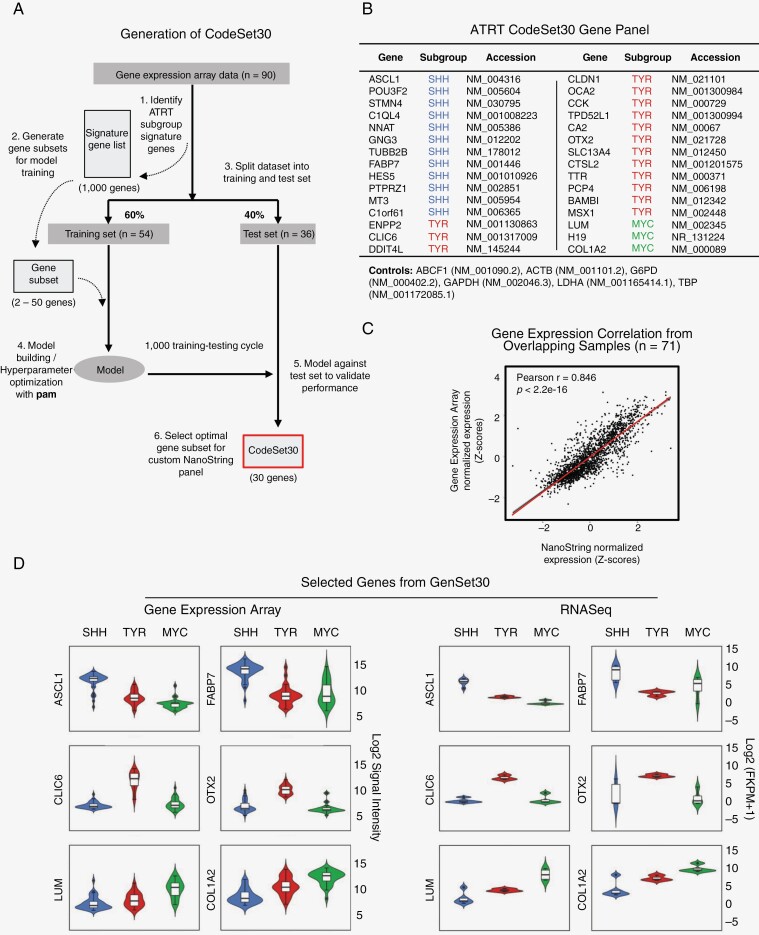
Development of CodeSet30 as ATRT signature gene set. (A) Schematic outline of how our ATRT subgroup-specific signature gene set is derived from gene expression data.^8^ For model training, significant differentially expressed ATRT subgroup genes (≥3-fold change, adj. *P* < .005) were used, with data randomly split into training set (60%) for PAM algorithm driven cross-validated training, and testing set (40%) for performance evaluation. Thirty optimal genes were selected (CodeSet30) for the NanoString probe set gene panel after repeating training-testing cycle. (B) Gene list of CodeSet30 to be used for custom NanoString ATRT subgrouping assay. (C) Correlation of normalized and scaled CodeSet30 genes between gene expression array and NanoString dataset (*R*^2^ = 0.85). Diagnonal line indicates smoothed conditional means with 95% confidence interval. (D) Violin plots of subgroup-specific CodeSet30 gene expression levels stratified by ATRT subgroups across expression array and RNASeq dataset. Mean expression levels of selected genes in SHH, TYR and MYC subgroups are indicated by blue, red, green boxes respectively. Expression scale is shown on the right side of each platform panel.

To construct an ATRT signature panel for subgrouping, we have utilized the published Illumina HT12 v4 array dataset published in Torchia et al. (*n* = 90).^5^ The 50 most differentially expressed genes (≥3-fold change and adj. *P* < .005) were selected from each subgroup as gene candidates. The dataset was randomly split into training (*n* = 60) and testing (*n* = 30) cohorts, and we utilized the PAMR algorithm to optimize for the gene list with the highest testing accuracy over 1000 cycles (Figure 1A). Next, to ensure the genes selected are representative of ATRT subgroups and are free from platform bias, we compared the expression patterns against the RNASeq dataset.^5^ The final 30 gene signature list (CodeSet30) was intentionally selected for interpretability and included redundancy to account for sample quality variations. Six housekeeping genes were included for reference gene normalization (Figure 1B). Probes were ordered and synthesized at NanoString manufacturer (NanoString Technologies, Inc., Seattle, WA, USA).

We applied the CodeSet30 panel to ATRT tumor samples collected through the Rare Brain Tumor Consortium and Registry (www.rarebraintumorconsortium.ca) with informed consent as per protocols approved by the Hospital for Sick Children. RNAs were extracted from ATRT patient samples from frozen tissue and formalin-fixed paraffin-embedded (FFPE) tissues origin (*n* = 93, including 11 replicates). Extra-cranial malignant rhabdoid tumors (MRT) were also collected for this study (*n* = 14 including 2 replicates). Both the legacy and XT chemistry were tested in this study.

We have constructed an *in silico* tumor classification tool, AClass, that performs gene quantification using the output from the NanoString nCounter Analysis System. It also performs reference gene normalization and quality control from the control probes and the quality score calculated from the housekeeping genes. Samples that did not pass the quality score threshold (<100) and were removed from the analysis.

**Figure 2.**
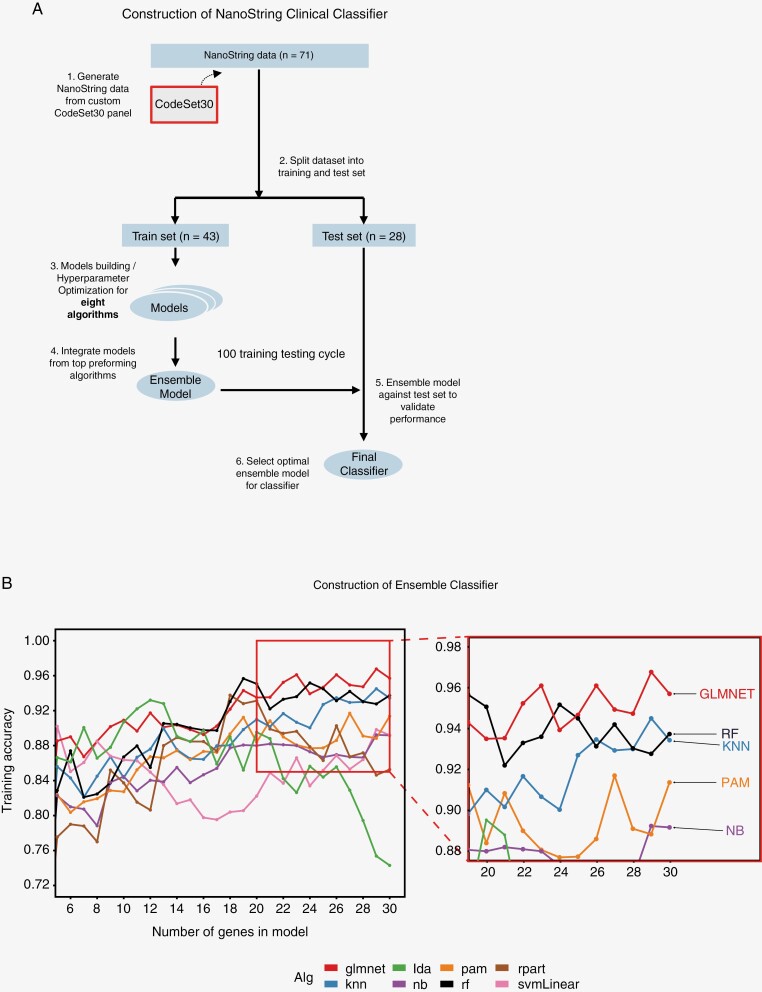
Model development using Ensemble classifier with NanoString platform. (A) Schema outlining our construction process of ATRT subgrouping ensemble classifier using NanoString data analysis. Data was split randomly into training (60%) and testing (40%) sets. The remaining test set was used to evaluate the classifier performance. After repeated training-testing process, the optimal ensemble model was selected as final classifier for analysis in this study. (B) Representative prediction score plot for eight algorithms: (x-axis) number of genes used in a model and (y-axis) training accuracies where 1 = perfect accuracy. To prevent overfitting, the top 5 best performing classification models from 8 algorithms (red box) were incorporated into an ensemble classifier. Average training accuracy of the top 5 algorithms: glmnet (0.950), knn (0.921), rf (0.937), pam (0.892) and nb (0.877).

To construct the NanoString ensemble classifier, we have tested eight diverse algorithms (glmnet, knn, rf, pam, nb, rpart, svmLinear, lda) across 2–30 genes (29 gene combinations) which produced a total of 232 models. Classifier performance was evaluated using samples with overlapping gene expression array subgroup labels (*n* = 71). Samples were split into 60% training and 40% testing with 5 repeats of 10-fold cross-validation. We have selected the top 5 algorithms with the highest average training accuracy and gene combinations—a total of 55 classification models—to create the final classification model using the same training/testing strategies as illustrated above (Figure 2A). A representative model trained using this method was used to classify both ATRT and MRT NanoString samples (see Supplementary Table 2 and Supplementary Methods for further information).

### CodeSet30 Validation and Performance Evaluation

The quality of the CodeSet30 panel is assessed by evaluating the ensemble classifier’s training/ testing accuracies. As an additional corroboration, we performed unsupervised multi-dimensional scaling analysis (MDS) to evaluate classification results.

To perform further validation on the validity of AClass, we verified the performance of CodeSet30 against the 26 gene list from Lersute et al.^9^ (Leruste26) using an Affymetrix HG-U133 Plus 2.0 expression dataset (affy) from ATRT Consensus study^8^ (*n* = 112). Noncoding genes from the CodeSet30 panel that are not available in the affy dataset were omitted. Ensemble model training/ testing using the affy dataset was performed as described above for the NanoString classifier.

We compared NanoString subgroups against DNA methylation subgroups and investigated possible factors contributing to the lower predictive scores. Tumor microenvironment and purity assessment were performed using methylCIBERSORT with an expanded stroma matrix for brain tumors (see Supplementary Methods for further information).

## Results

### Construction of the NanoString Classifier With CodeSet30 ATRT Signature Gene Set

To construct a tumor classifier for ATRT subgrouping, we first sought to generate a robust signature panel using existing primary ATRT gene expression array data (Figure 1A). The gene expression dataset of 90 primary ATRTs from Torchia et al.^5^ was analyzed to identify the most significant and differentially expressed genes in each ATRT subgroup. Specifically, we evaluated the top highly differentiated expressed genes (Supplementary Figure 1A) using the prediction analysis for microarrays (PAM) algorithm in a repeated training-testing process (Supplementary Figure 1B) and identified genes that were exclusively expressed in a particular subgroup but were under-expressed in the rest of the subgroups. A final panel of 30 genes was selected to be used as the ATRT signature gene set (CodeSet30). The panel comprises unique gene expression patterns that can be used as markers for the 3 defined ATRT subgroups (Figure 1B). In particular, 12 overexpressed genes were chosen for the ATRT-SHH subgroup, 15 and 3 genes mapping to TYR and MYC subgroups, respectively. The expression level of CodeSet30 genes between NanoString data and gene expression array was highly correlated (Pearson *R* = 0.846, *P* < 2.2–16, Spearman *R* = 0.845) (Figure 1C), corroborating the utilization of CodeSet30 to define ATRT subgrouping. Most importantly, these 30 genes were able to retain subgroup-specific gene expression patterns across external gene expression array and RNASeq data (Figure 1D). Principal component analysis with gene expression and RNASeq data also indicate 3 distinct clusters corresponding to published subgroup labels (Supplementary Figure 1C). Collectively, our analysis results show that CodeSet30 confers a robust signature gene panel for ATRT subgroup stratification.

Next, we sought to construct an ATRT-specific NanoString classifier that implements ensemble classification and feature construction methods to mitigate the challenges of data overfitting with a relatively small sample size (Figure 2A). The main advantage of using the ensemble approach is that aggregation of a large number of predictions overcomes the biases and variances of using single algorithms, and in turn, improves the overall prediction. To accomplish this, a total of eight diverse algorithms were evaluated to select the top-performing algorithms for ATRT subgroup prediction based on the published gene expression subgroup labels.^5^ In the end, the top 5 algorithms with the highest average training accuracy and 11 gene combinations resulting in a total of 55 models, were incorporated into the final classifier. The final subgroup identification of a sample would be the one that is favored by the majority of the 55 models. The prediction score represents the average class prediction probabilities of the models voted for the same subgroup. Using this method, we observed the overall average training accuracy to be 91.9% for our ATRT NanoString classifier, which we named ATRT Classifier (AClass) (Figure 2B).

### Evaluation of ATRT Classifier Subgroup Prediction Performance

**Figure 3.**
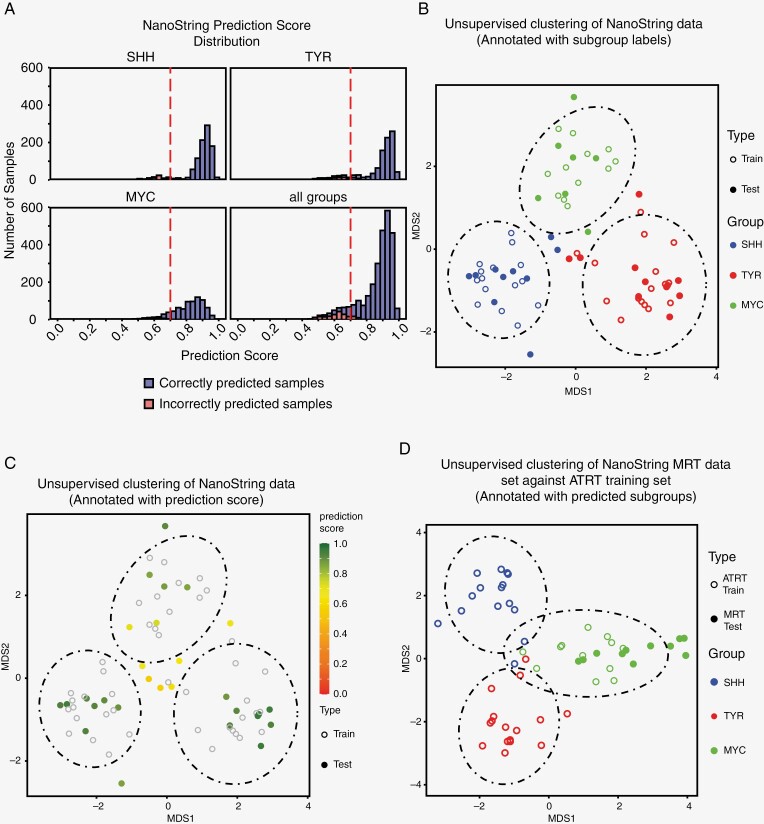
Evaluation of NanoString classifier’s performance on stratifying ATRT. (A) Distribution of binned classifier prediction scores from all combinations of training and testing samples in validation set (Related to Table 1). Prediction scores from correctly predicted samples (blue) were compared to incorrectly predicted samples (red) within each subgroup and across all samples. The prediction score threshold at 0.7 (red dotted line) was determined by maximizing the proportion of accepted samples and minimizing the rejection rate. (B, C) Unsupervised clustering of NanoString data from test samples (solid circle) against training samples (open circle) by multi-dimensional scaling analysis (MDS). Data annotated with (B) predicted subgroup labels (red = SHH, blue = TYR, green = MYC) and (C) prediction score in color gradient. Dotted ellipses = 95% confidence interval with respect to the centroid for training samples in each subgroup. (D) MDS analysis of 11 MRT (solid circle) against ATRT training set (open circle). Training labels and testing results are color coded based on subgroups (red = SHH, blue = TYR, green = MYC). The dotted ellipses represent the 95% confidence interval for training samples in each subgroup.

To evaluate the robustness of AClass, we performed training and testing on the validation set of 30 samples for model prediction accuracy in 10 independent trials. We observed an average accuracy of 93.6% with a coefficient of variation of 2.3%. When stratifying the validation set of these 10 independent trials by their ATRT subgroups, both SHH and TYR subgroups produced a narrow distribution of correctly predicted samples with high-median prediction scores of 0.913 and 0.920, respectively, while the MYC subgroup produced a wider prediction score distribution with a relatively lower median prediction score of 0.827 (Figure 3A). To maximize classification specificity for clinical usage, we applied a prediction threshold of 0.7 to disqualify any sample with a low prediction score. This threshold is determined based on the validation set prediction scores distribution (Figure 3A). With the prediction threshold, the average accuracy increased to 98.9% (Table 1) with an average of 12.5% of the samples filtered due to a lower prediction score (“rejection rate”). Refer to Supplementary Table 1 for detailed performance metrics.

**Table 1.** Prediction Summary for Training and Testing Runs Relative to Prediction Threshold

Independent run #		Validation Set Classification Results			
	Prediction Threshold	0.0		0.7	
		Accuracy	Reject. Rate	Accuracy	Reject. Rate
1		89.0%	0.0%	96.0%	11.0%
2		93.0%	0.0%	100.0%	18.0%
3		93.0%	0.0%	100.0%	7.0%
4		96.0%	0.0%	100.0%	14.0%
5		96.0%	0.0%	100.0%	7.0%
6		93.0%	0.0%	100.0%	18.0%
7		93.0%	0.0%	100.0%	14.0%
8		96.0%	0.0%	100.0%	18.0%
9		93.0%	0.0%	92.0%	11.0%
10		93.0%	0.0%	100.0%	7.0%

To further evaluate the prediction performance of AClass, we performed unsupervised multi-dimensional scaling (MDS) clustering analysis on the normalized NanoString data. We observed that samples with high-prediction scores fell into clusters that correspond to the ground truth labels^5^ with an absence of batch effect, suggesting that prediction from AClass tightly matches with subgrouping results based on unsupervised gene expression clustering. On the other hand, samples with low prediction scores were drawn away from the 3 main clusters and lay in the center of the plot or at the main clusters’ peripherals (Figure 3C). As such, we have demonstrated that the AClass ensemble classification approach is suitable for ATRT subgrouping.

Given that extra-cranial malignant rhabdoid tumors (MRT) also cluster with ATRT’s as alluded to in several studies,^5,11^ we investigated whether AClass would also be applicable to stratify MRT. We collected 14 MRTs from various body locations including kidneys and other soft tissues. Out of the 14 MRTs, 3 samples did not pass the quality threshold and were removed from the analysis. Using AClass with pretrained ATRT models, all 11 MRTs were classified as MYC. Unsupervised MDS analysis of the ATRT training samples and MRT were also performed, and results corroborated with the classifier prediction (Figure 3D). Details of each of the MRT samples and the classification results can be found in Supplementary Table 2. Together, our aggregate data suggests that the AClass NanoString classifier would also be suitable for both ATRT and MRT molecular subgroup stratification.

### Comparison of CodeSet30 and Other ATRT Biomarkers

Recently, Leruste et al. have published a custom 26 genes NanoString panel (Leruste26) for tumor subgrouping of 45 FFPE ATRTs using an unsupervised clustering method and compared against subgroups labels from the RNASeq dataset.^9^ The 26 most discriminant genes were selected from a published Affymetrix Ul33 plus2 (affy) gene expression dataset consisting of 92 published samples. Comparison between CodeSet30 and Leruste26 revealed minimal overlap between the signature gene panels with only 6 genes overlapping: 2 in SHH, 4 in TYR, and none in the MYC subgroup (Supplementary Table 3).

**Figure 4.**
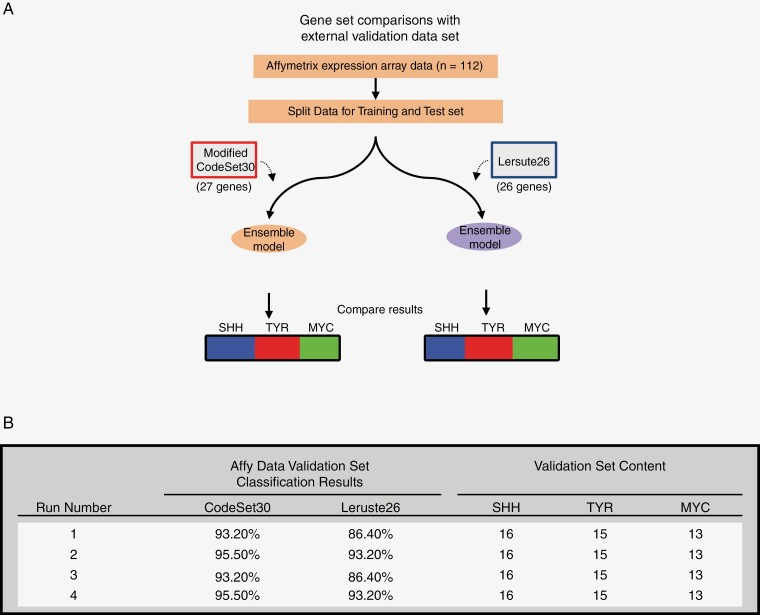
Comparative NanoString classifier modeling between CodeSet30 and other ATRT code sets. (A) Schematic outline of Ensemble classifier retraining process using Affymetrix expression array dataset of 112 primary ATRT tumors. Data was split randomly into training (60%) and testing (40%) sets. At least 4 training-testing cycles were performed. (B) Gene set performances of Modified CodeSet30 and Leruste26 were evaluated by comparing the classification results from identical training/ validation sets.

We therefore sought to compare the performance of CodeSet30 against Leruste26 in ATRT subgrouping using an independent ATRT dataset. First, we retrained the NanoString classifier with a published affy gene expression dataset from 112 ATRT samples and their subgroup assignments used in the ATRT consensus study.^8^ Model training and testing were performed as described earlier with CodeSet30 except 3 noncoding genes (*C1QL4, CTSL2,* and *H19*) were not included in the affy dataset (Human Genome U133 Plus 2.0 Assay) and were therefore omitted (Figure 4A). Results from the randomized training and testing trials showed an average prediction accuracy ranging between 93.2% and 95.5% in 4 independent trials of training and validation, without any prediction score threshold (Supplementary Table 4). Next, to allow direct comparisons between CodeSet30 and Leruste26, we performed randomized training and testing with identical samples for both gene sets. We observed that GeneCode30 consistently achieved higher accuracy in all 4 runs compared to Leruste26 (average accuracy of 94.3% and 89.8% respectively, Figure 4B). Thus, we have successfully tested the performance of CodeSet30 using an independently generated affy dataset and against Leruste26. Notably, the accuracies obtained from the affy dataset were comparable to the NanoString dataset, indicating that the gene set in CodeSet30 could be applicable for ATRT subgrouping across different transcriptomic platforms.

During our analysis process, we also evaluated RNA sample quality between frozen tissues and FFPE derived from the same tumors (Supplementary Figure 2). As expected, FFPE samples obtained a much lower average quality score compared to frozen samples (average quality score 224 and 5414, respectively). In particular, included in our NanoString dataset were samples from patient RBTC427, which had both frozen tissue and FFPE samples. We observed heavy degradation in RNA integrity as evident from the quality score of 6880 in the fresh frozen sample compared to 224 in the FFPE counterpart. Consistent with gene expression array and methylation clustering classification results, both samples were classified as the same subgroup as TYR, albeit with a lower confidence score in the FFPE sample (0.93 compared to 0.60). Our data also showed that the quality of the samples correlates with the samples’ age as suggested by Northcott et al.^10^ (Supplementary Figure 2).

To investigate the performance between frozen and FFPE samples, we have further generated 2 collections of ATRT on the NanoString platform and analyzed them with AClass workflow using the same processing settings (Quality Score threshold ≥100, Prediction Score ≥0.7). As specimens were retrospectively collected from medical facilities around the World, we expected there to be variations in the classification outcome as fixation conditions (eg fixation time and formalin used) and storage conditions (eg, temperature and humidity) were not known.

The first dataset is made up of ~40% of FFPE and frozen samples and all the samples passed the quality threshold. There is a higher proportion of high-confidence prediction in the frozen samples compared to FFPE (avg. prediction score 0.855 vs 0.770 respectively). The second data set is mostly comprised of archival samples with ~75% being FFPE samples. Ninety percentage of the samples passed the quality threshold with all the samples that have poor quality samples being FFPE tissues. Here, frozen samples have a slightly higher proportion of high-confidence prediction than FFPE (avg. prediction score was 0.857 vs 0.827, respectively). We noted that, while frozen samples performance is relatively stable, FFPE samples in general achieved lower and more varied prediction score and model agreement due to factors that can be difficult to control for. Due to these reasons, the same threshold has been applied to both frozen and FFPE samples in this study. We demonstrated the feasibility of subgrouping old archival samples but also noted that adjustment to acceptance criteria for FFPE samples (such as for borderline cases) is also valid and subject to individual sample assessment.

### Comparison of Tumor Subgrouping With AClass and DNA Methylation Brain Tumor Classifier

**Figure 5.**
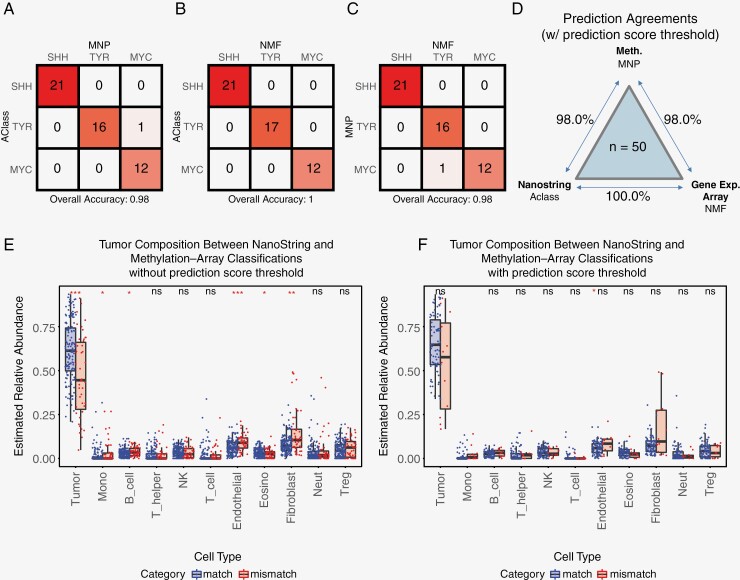
Classification alignment of AClass with gene expression array and DNA Methylation Brain Tumor Classifier (A–C) Confusion matrix of 50 samples comparing classification results from gene expression array (NMF), DNA methylation (MNP) and NanoString profiling (AClass). All samples passed the acceptance threshold from the corresponding algorithm. (D) Prediction concordances between the 3 platforms tested in this study. (E and F) Boxplot of the relative proportion of immune infiltration levels in samples with both NanoString and methylation array data as predicted by MethylCIBERSORT. All samples with NanoString and methylation array prediction are stratified by matching or mismatched ATRT subgroup prediction. Levels of tumor content and immune-cell types infiltration were compared (E) across all samples without applying prediction score and (F) for samples that passed the acceptance threshold. *P ≤ .05, **P ≤ .01, ***P ≤ .001, “ns” *P* > .05.

The DNA methylation-based CNS tumor classifier (www.molecularneuropathology.org, hereinafter “MNP”) from Capper et al. has been demonstrated to be a widely popular and useful tool for ATRT subgrouping and has been utilized in multiple ATRT clinical studies.^7,12–14^ We sought to investigate the agreement between the NanoString transcriptomic subgrouping against the DNA methylomic subgrouping. To that end, ATRT samples used for AClass construction with sufficient materials were analyzed using Illumina DNA methylation array, and ATRT subgroups were predicted using MNP. Results from AClass showed an 88.7% (55/62) agreement with MNP. When we applied the prediction score threshold of 0.7 to AClass and 0.9 to the calibrated score from MNP (as per recommendation^12,15^), agreement increased to 98.0% (49/50) (Figure 5A, Supplementary Figure 5). Expectedly, when comparing the 3 commonly used tumor subgrouping platforms, the 2 RNA-based platforms showed higher agreement as compared to that between RNA and DNA methylation-based platforms (Figure 5B–D). Our results indicated that the molecular subgroupings mostly remain consistent for high-confidence samples regardless of the method.

To investigate potential factors affecting the agreement between transcriptomic and methylation-based classification methods, we utilized the cellular deconvolution algorithm Methyl-CYBERTSORT with a brain-specific reference set. This analysis allows us to infer the proportion of tumor contents and immuno-cellular cells based on each sample’s DNA methylation profile. For samples with mismatched subgroup classification between AClass and MNP, we observed significantly lower tumor purity (*P* < .001) as reflected in the elevated proportion of endothelial cells and fibroblasts, along with higher levels of immuno-cellular contents (such as monocytes and B cells). We further observed that applying the prediction score threshold can effectively remove low purity samples (Figure 5E and F). This suggested that tumor heterogeneity contributes to lower prediction scores and disagreement between subgroup predictions.

### Deployment and Reproducibility of AClass Workflow

To facilitate the utilization of the AClass workflow, we have outlined a clinical workflow schema from laboratory sample processing to data analysis based on our experience at the NanoString facility at the Hospital for Sick Children (Supplementary Figure 3A). This workflow utilizes the AClass algorithm using pretrained CodeSet30 models to ensure consistency and reproducibility. From our experience, analysis results can be obtained for fresh frozen tumors and archival FFPE biopsies with minimal bioinformatics support (Supplementary Figure 3B) and a fraction of the time and cost compared to the DNA methylation array (Supplementary Table 7).

During the development of our assay, an updated NanoString reagent Extension TagSet (XT) was released. Therefore, we sought to repeat our experiment with 11 samples to investigate whether the XT reagent would yield the same prediction outcome using the same NanoString probes. Comparative analysis showed a high-correlation between the prediction scores (*R*^2^ = 0.96, *P* = 3.33e−06) using the legacy chemistry reagent and the XT reagent (Supplementary Figure 4A). Furthermore, assays using the 2 reagents yielded identical classification results (Supplementary Figure 4B), suggesting that the difference in NanoString reagents would not alter the robustness of the AClass NanoString classifier result.

## Discussion

In recent years, the heterogeneous molecular features and clinical outcomes of ATRTs are increasingly being recognized in the hopes of providing more personalized treatment plans. Yet, there has been an absence of tools that allow rapid and affordable stratification of ATRT patients by their molecular subgroup. While array-based unsupervised clustering is useful for subgroup discovery, the supervised subgroup classification method provides consistent results based on the training model and would require only the essential features for classification—saving both cost and time. A major hurdle in developing an ATRT subgroup classifier was the rarity of ATRT samples because classification algorithms tend to be subject to overfitting under low sample numbers. Moreover, as formalin-fixation and paraffin-embedding (FFPE) are often the methods of choice for preserving clinical tumor specimens, there is a need for a reliable and affordable ATRT classifier compatible with archival tissue samples. To that end, we have developed a readily applied classifier (AClass) that implements an ensemble of algorithms with well-characterized ATRT samples. By combining multiple algorithms with different modalities of the data, the ensemble strategy has been demonstrated to outperform the single-algorithm method in terms of accuracy and robustness.^16^ We demonstrated that CodeSet30 and AClass perform well on multiple gene expression platforms including microarray, RNASeq, and NanoString. Using the NanoString platform, a proven reliable clinical platform for developing tumor classification tools,^10^ we demonstrated that our workflow enables the analysis of RNA samples obtained from fresh frozen or FFPE biopsies of primary tumors.

Performance based on our NanoString validation data shows high accuracy and sensitivity (Supplementary Table 1). In addition, we have used an independently published ATRT Affymetrix expression array dataset to validate our method against a recently published NanoString gene set from Leruste et al.^9^ For this comparison, we have separately retrained our ensemble classifier using CodeSet30 and Leruste26 gene set using the affy dataset and observed that the overall accuracies between the 2 gene sets are highly comparable from 4 separate training and testing processes, with GeneCode30 achieving higher accuracy in all runs (Supplementary Table 4). Leruste26 gene set was used for *unsupervised* clustering in the original publication and our analysis has shown that its gene list can also be repurposed for *supervised* classification with AClass. This demonstrates the flexibility of our workflow and the potential to reanalyze previously published data using AClass. When comparing the 2 gene lists, it is apparent that the selected genes largely serve similar biological roles suggesting concordance in cellular regulatory networks. Within the SHH subgroup, several genes are related to neurogenesis (*ASCL1*, *GLI2*, and *POU3* in the TYR subgroup, genes often involved in cell proliferation (*ENPP2*, *TPD52L1*) and genes highly expressed in the retina (*CLIC6*, *SLC13A4*). While there are no overlapping genes in the MYC subgroup, genes are typically structural-related, such as in cytoskeleton remodeling (*PRPH*) and fibrillar collagen organization (*LUM*, *COL1A2*). All in all, many of these genes are in line with gene expression profiles from earlier studies.^5,8^ The choice of transcriptomic platforms used to generate signature gene lists impacts the choice of signature genes, due to the inherent design biases and detection limitations of each technology.

Close evaluation of the incorrectly classified samples revealed that there were consistently fewer misclassifications using CodeSet30 prediction (2 misclassified samples) as compared to Leruste26 prediction (3–6 misclassified samples).

Both gene sets had a roughly equal ratio of misclassifications falling within the SHH and MYC clusters. Interestingly, when classifiers trained from both gene sets were wrong, they had identical class predictions. This is likely due to the similarity in biological functions of the selected genes. Finally, despite Lesrute26 having relatively more MYC subgroup signature genes, this subgroup still makes up almost half of the misclassified samples (Supplementary Table 5). This highlights the heterogeneous nature of the MYC subgroup.

In our study, all 11 of the MRT’s were classified as MYC subgroups despite varied tumor locations (Supplementary Table 2). It is worth noting that MRT’s are mostly classified as MYC subgroups although not exclusively, with 1 study reporting 85% of the extra-CNS MRT using the methylation array platform.^17^ This preferential subgroup bias by tumor location is consistent with previous research on human ATRT where SHH was suspected to be of neural-origin and MYC was suspected to be of mesenchymal-origin.^4,11^

Our study showed that sample quality is one of the key determinants of prediction accuracy. Tumor sample impurity is sometimes inevitable with archived specimens, and it can be mitigated by applying a prediction threshold in AClass classification. The typical harsh FFPE tissue processing process is known to contribute to the low RNA quality resulting in sequencing artifacts that limit their use for molecular analysis.^10,18^

While both transcriptomic and DNA methylomic-based classification methods have been used in literature for ATRT subgrouping, there lack of a comparison between the 2 methods. Limited by the rarity of ATRT, an extensive comparison is proven to be difficult. Nonetheless, we strive to perform a small-scale investigation using 58 samples from our study to be analyzed using the DNA methylation brain tumor classifier from Capper et al. Results from our transcriptome-based ensemble classifier showed a concordance rate of 84.48% (49/58) and up to 95.83% (46/48) when applying the prediction score threshold (Supplementary Figure 6). This result is comparable to Korshunov et al.’s reported discrepancies between NanoString and methylation classification results for 239 medulloblastoma (MB) samples.^19^ Comparing the results from RNA-based against DNA methylomic-based classifiers, Korshunov et al. reported 8% of subgroup switching and 8% of non-MB were identified.^19^ We have investigated several factors that would lead to disagreement and have identified several possible contributing factors. First, our analysis based on the cellular component deconvolution analysis method MethylCIBERSORT suggests that the discrepancy between ATRT RNA-based and DNA methylomic-array-based methods could be due to lower tumor purity and immune infiltration. Even after applying stringent prediction score filtering, there is still an elevated level of monocytes in samples that had mismatched prediction results (Figure 5F). Another possible source of discrepancy might result from the MYC group being less well defined, as it encompasses rhabdoid tumors in every CNS location and soft tissue, as in the case of MRT. Unlike ATRT, MRTs tend to harbor unstable genomes.^17^ MYC group’s heterogeneity contributes to less distinct signature genes for this subgroup, as reflected in our panel, which renders it less specific than the other 2 subgroups. Further subdivisions within the existing subgroups may also contribute to this disagreement.^4,5,7^ A recent study on *SMARCA4* deficient ATRTs by Holdhof et al. reports that the DNA methylation classifier classified 50% of the 14 samples as an SHH, 21% without a specific match with ATRTs, and the remaining 29% not match methylation class at all.^20^ This study has several implications. First, the DNA methylation classifier can be limited by the subgroup labels and the tumor samples the models were trained with. It is still important to include unsupervised analysis as an evaluation method, such as MDS analysis as we have implemented in AClass when assessing subgroup results.

We wish to illustrate here some practical considerations of applying AClass, with emphasis comparing with the DNA methylation array requirements illustrated by Heidelberg et al.^12^ The reliability, fast turnaround time, and relatively lower cost are the advantages of the NanoString workflow. The turnaround time is estimated to be within 2 days (as much as 10 days for DNA methylation array) with one-tenth of the cost (Supplementary Table 7) for FFPE samples and about one-eight for frozen samples. In terms of sample input, both techniques are compatible with FFPE samples. From our experience, NanoString is more tolerant to low input amounts and does not require the extra steps of bisulphite conversion and FFPE DNA restoration. With CodeSet30’s simple gene panel design (vs thousands of CpG sites required by MNP) and usage of targeted probes, we have good results with 100 ng of samples using the NanoString platform (vs DNA methylation array that uses >500 ng fresh frozen samples and 250 ng of DNA for most FFPE tissues). Importantly, this study also shows that the 2 methods can complement each other, as seen from failed samples between the 2 methods typically do not overlap (Supplementary file). The downside to AClass is that it is solely aimed at ATRT classification. It does not allow copy number variant inference like the DNA methylation array. CodeSet30 is also not designed to differentiate ATRT from other tumor entities. Despite the prediction threshold in principle can remove samples that do not resemble the 3 ATRT subgroups, AClass is only intended for ATRT subgrouping and not a tool for confirmation of ATRT entity, which should be established prior to running AClass. Expansion of the NanoString probe set would be possible with retraining models with appropriate samples.

As for the clinical utility of ATRT subgrouping, the current opinion is that ATRT could be associated with distinct therapeutic vulnerabilities. For example, a growing number of studies show that the MYC subgroup is sensitive to TKI treatments using in vitro and in vivo models.^5,21,22^ Also, significant immune-cell infiltration has been reported in MYC and TYR tumors,^9,11^ which suggests that immune checkpoint inhibition could be a potential therapeutic strategy for these tumors. The prognostic value of the ATRT molecular subgroup has been analyzed in the prospective clinical trial from the Children’s Oncology Group (COG) ACNS0333 trial^1^ and the retrospective study European Rhabdoid Registry (EU-RHAB) study^13^ and by colleagues from St. Judes^13^ but with varying findings. The EU-RHAB and St Judes cohort identified the non-TYR profile as an independent negative prognostic marker. Whereas the COG trial found a much more favorable prognosis for patients with SHH tumors with a 6-month EFS of 100%. Thus, the impact of molecular grouping on patient outcomes awaits further validation.

In summary, we have demonstrated the flexibility of our classifier by achieving 94% testing accuracy using an independently acquired gene expression dataset (CodeSet30) and have shown that our gene set achieved better accuracy against other published gene sets. The AClass classification method was designed to allow for flexibility in the choice of gene set and data platform source. In this study, we have demonstrated its use for NanoString and gene expression array data. Compared to DNA methylation array and RNASeq, NanoString technology does not involve lengthy sample preparation procedures and is compatible with FFPE tissues, making it an ideal solution for tumor subgrouping. This study has demonstrated a practical, rapid, and affordable ATRT subgrouping workflow. From building upon findings in ATRT subgroups, our classifier facilitates providing molecularly tailored therapy for patients in each of the subgroups and translating research discoveries to clinical practice.

## Supplementary Material

vdae004_suppl_Supplementary_Tables_S1-S7

vdae004_suppl_Supplementary_Data

vdae004_suppl_Supplementary_Figures_S1-S6

## Data Availability

NanoString data are deposited at the European Genome-Phenome Archive, EGA Study Accession ID EGA: EGAS00001007470.
