# Supplementary material for: Rapid, economical diagnostic classification of ATRT molecular subgroup using NanoString nCounter platform
Source: Neurooncol Adv. 2024 Jan 16;6(1):vdae004. doi: 10.1093/noajnl/vdae004 (PMC10825849; doi:10.1093/noajnl/vdae004)
Supplement: vdae004_suppl_Supplementary_Tables_S1-S7 [file vdae004_suppl_supplementary_tables_s1-s7.docx]

Table S1 – AClass performance metrics from repeated k-fold cross validation (as described in Fig. 2A)

|  |  |  |  |  |  |  |  |  |  |  |  |  |  |  |
| --- | --- | --- | --- | --- | --- | --- | --- | --- | --- | --- | --- | --- | --- | --- |
|  | Without prediction score threshold | |  |  |  |  |  |  |  |  |  |  |  |  |
|  | Class | Probability Threshold | Sensitivity | Specificity | Pos Pred Value | Neg Pred Value | Precision | Recall | F1 | Prevalence | Detection Rate | Detection Prevalence | Balanced Accuracy |  |
|  | SHH | 0 | 0.94 | 0.95 | 0.924498834 | 0.968654971 | 0.9244988 | 0.94 | 0.9271122 | 0.357142857 | 0.335714286 | 0.367857143 | 0.945 |  |
|  | TYR | 0 | 0.92 | 0.988888889 | 0.981818182 | 0.959398496 | 0.9818182 | 0.92 | 0.9465133 | 0.357142857 | 0.328571429 | 0.335714286 | 0.954444444 |  |
|  | MYC | 0 | 0.9375 | 0.96 | 0.911944444 | 0.975884028 | 0.9119444 | 0.938 | 0.9201424 | 0.285714286 | 0.267857143 | 0.296428571 | 0.94875 |  |
|  |  |  |  |  |  |  |  |  |  |  |  |  |  |  |
|  | With prediction score threshold | |  |  |  |  |  |  |  |  |  |  |  |  |
|  | Class | Probability Threshold | Sensitivity | Specificity | Pos Pred Value | Neg Pred Value | Precision | Recall | F1 | Prevalence | Detection Rate | Detection Prevalence | Balanced Accuracy |  |
|  | SHH | 0.700 | 1.000 | 1.000 | 1.000 | 1.000 | 1.000 | 1.000 | 1.000 | 0.366877456 | 0.366877456 | 0.366877456 | 1 |  |
|  | TYR | 0.700 | 0.979 | 1.000 | 1.000 | 0.989 | 1.000 | 0.979 | 0.989 | 0.350152514 | 0.342734932 | 0.342734932 | 0.989444444 |  |
|  | MYC | 0.700 | 1.000 | 0.989 | 0.978 | 1.000 | 0.978 | 1.000 | 0.988 | 0.28297003 | 0.28297003 | 0.290387612 | 0.994722222 |  |

Table S2 – Nanostring CodeSet30 classification of extra-cranial malignant rhabdoid tumors

|  |  |  |  |  |  |  |
| --- | --- | --- | --- | --- | --- | --- |
|  |  |  | Classification Results | | |  |
|  | Sample Id | Tumor Location | Quality Score | ATRT  Subgroup | Prediction Score |  |
|  |  |  |  |  |  |  |
|  | RBTC1278 | Kidney | 1348.24 | MYC | 0.9 |  |
|  | RBTC1279 | Kidney | 1302.19 | MYC | 0.82 |  |
|  | RBTC1288 | Kidney | 2145.81 | MYC | 0.92 |  |
|  | RBTC1289 | Head and Neck | 209.53 | MYC | 0.82 |  |
|  | RBTC1290 | Extemity | 8592.91 | MYC | 0.93 |  |
|  | RBTC1294 | Abdomen | 1004.53 | MYC | 0.92 |  |
|  | RBTC1295 | Chest wall / throax | 8860.83 | MYC | 0.93 |  |
|  | RBTC1296 | Abdomen | 5982.72 | MYC | 0.93 |  |
|  | RBTC1298 | Bladder | 1347.68 | MYC | 0.89 |  |
|  | RBTC1301 | Kidney | 1431.43 | MYC | 0.92 |  |
|  | RBTC1302 | Kidney | 3882.53 | MYC | 0.93 |  |
|  |  |  |  |  |  |  |

Table S3 – Comparison of CodeSet30 and Leruste26

|  |  |  |  |  |  |  |  |  |
| --- | --- | --- | --- | --- | --- | --- | --- | --- |
|  | Gene sets used in the study | | | | | | |  |
|  | CodeSet30 | | |  | Leruste26 | | |  |
|  | Gene |  | Subgroup |  | Gene |  | Subgroup |  |
|  |  |  |  |  |  |  |  |  |
|  | ASCL1 | a | SHH |  | ASCL1 | a | SHH |  |
|  | POU3F2 | a | SHH |  | POU3F2 | a | SHH |  |
|  | STMN4 |  | SHH |  | OSBPL6 |  | SHH |  |
|  | C1QL4 |  | SHH |  | KIF21A |  | SHH |  |
|  | NNAT |  | SHH |  | PSD3 |  | SHH |  |
|  | GNG3 |  | SHH |  | GLI2 |  | SHH |  |
|  | TUBB2B |  | SHH |  | CLIC6 | a | TYR |  |
|  | FABP7 |  | SHH |  | TRPM3 |  | TYR |  |
|  | HES5 |  | SHH |  | ENPP2 | a | TYR |  |
|  | PTPRZ1 |  | SHH |  | SNX25 |  | TYR |  |
|  | MT3 |  | SHH |  | TPD52L1 | a | TYR |  |
|  | C1orf61 |  | SHH |  | MITF |  | TYR |  |
|  | ENPP2 | a | TYR |  | SLC13A4 | a | TYR |  |
|  | CLIC6 | a | TYR |  | SLC6A20 |  | TYR |  |
|  | DDIT4L |  | TYR |  | CYB5R2 |  | TYR |  |
|  | CLDN1 |  | TYR |  | STRIP2 |  | TYR |  |
|  | OCA2 |  | TYR |  | CALML4 |  | TYR |  |
|  | CCK |  | TYR |  | HTR2C |  | TYR |  |
|  | TPD52L1 | a | TYR |  | TYR |  | TYR |  |
|  | CA2 |  | TYR |  | PRPH |  | MYC |  |
|  | OTX2 |  | TYR |  | SHC1 |  | MYC |  |
|  | SLC13A4 | a | TYR |  | ADGRE5 |  | MYC |  |
|  | CTSL2 |  | TYR |  | PLOD1 |  | MYC |  |
|  | TTR |  | TYR |  | RSU1 |  | MYC |  |
|  | PCP4 |  | TYR |  | TNFRSF1A |  | MYC |  |
|  | BAMBI |  | TYR |  | SEC24D |  | MYC |  |
|  | MSX1 |  | TYR |  |  |  |  |  |
|  | LUM |  | MYC |  |  |  |  |  |
|  | H19 |  | MYC |  |  |  |  |  |
|  | COL1A2 |  | MYC |  |  |  |  |  |
|  |  |  |  |  |  |  |  |  |

^a^Gene found in both CodeSet30 and Leruste26

Table S4 – ATRT subgroup classification result on Affymetrix dataset using CodeSet30 and Leruste26

|  |  |  |  |  |  |  |  |  |  |
| --- | --- | --- | --- | --- | --- | --- | --- | --- | --- |
|  |  |  | Affy Data Validation Set Classification Results | |  | Validation Set Content | | |  |
|  |  |  |  |  |  |  |  |  |  |
|  | Run Number |  | CodeSet30 | Leruste26 |  | SHH | TYR | MYC |  |
|  |  |  |  |  |  |  |  |  |  |
|  | 1 |  | 93.20% | 86.40% |  | 16 | 15 | 13 |  |
|  | 2 |  | 95.50% | 93.20% |  | 16 | 15 | 13 |  |
|  | 3 |  | 93.20% | 86.40% |  | 16 | 15 | 13 |  |
|  | 4 |  | 95.50% | 93.20% |  | 16 | 15 | 13 |  |
|  |  |  |  |  |  |  |  |  |  |

Table S5 – supplemental to Table 1. Discarded sample prediction from using CodeSet30 and Leruste26

|  |  |  |  |  |  |  |  |  |  |  |  |
| --- | --- | --- | --- | --- | --- | --- | --- | --- | --- | --- | --- |
|  |  |  |  | Incorrectly Classified Samples | | | | | | |  |
|  |  |  |  | CodeSet30 | | |  | Leruste26 | | |  |
|  | Run Number | Sample Id | Ground Truth | ATRT Subgroup | Prediction Score | Models Agreement |  | ATRT Subgroup | Prediction Score | Models Agreement |  |
|  |  |  |  |  |  |  |  |  |  |  |  |
|  | 1 | dkfz0592 | SHH |  |  |  |  | MYC | 0.8 | 0.7 |  |
|  |  | gsm1562910 | SHH |  |  |  |  | MYC | 0.75 | 0.66 |  |
|  |  | gsm1562911 | SHH | MYC | 0.72 | 0.53 |  | MYC | 0.78 | 0.86 |  |
|  |  | rb06arn0010s | SHH |  |  |  |  | MYC | 0.72 | 0.66 |  |
|  |  | gsm1562925 | MYC |  |  |  |  | SHH | 0.8 | 0.76 |  |
|  |  | gsm1562934 | MYC | SHH | 0.81 | 0.86 |  | SHH | 0.75 | 0.74 |  |
|  | 2 | dkfz0419 | TYR | MYC | 0.79 | 0.51 |  | MYC | 0.76 | 0.77 |  |
|  |  | gsm1562928 | MYC |  |  |  |  | SHH | 0.85 | 0.95 |  |
|  |  | gsm1562934 | MYC | SHH | 0.81 | 0.83 |  | SHH | 0.68 | 0.54 |  |
|  | 3 | dkfz0592 | SHH |  |  |  |  | MYC | 0.8 | 0.7 |  |
|  |  | gsm1562910 | SHH |  |  |  |  | MYC | 0.77 | 0.65 |  |
|  |  | gsm1562911 | SHH | MYC | 0.72 | 0.52 |  | MYC | 0.78 | 0.86 |  |
|  |  | rb06arn0010s | SHH |  |  |  |  | MYC | 0.7 | 0.71 |  |
|  |  | gsm1562925 | MYC |  |  |  |  | SHH | 0.81 | 0.76 |  |
|  |  | gsm1562934 | MYC | SHH | 0.81 | 0.86 |  | SHH | 0.76 | 0.73 |  |
|  | 4 | dkfz0419 | TYR | MYC | 0.77 | 0.52 |  | MYC | 0.75 | 0.63 |  |
|  |  | gsm1562921 | TYR | MYC | 0.77 | 0.45 |  |  |  |  |  |
|  |  | dkfz1710 | MYC |  |  |  |  | SHH | 0.63 | 0.6 |  |
|  |  | gsm1562925 | MYC |  |  |  |  | SHH | 0.84 | 0.98 |  |
|  |  |  |  |  |  |  |  |  |  |  |  |

Table S6 – Summary of fresh frozen and FFPE datasets classification results

|  |  |  |  |  |  |  |  |  |  |  |  |  |  |  |  |  |
| --- | --- | --- | --- | --- | --- | --- | --- | --- | --- | --- | --- | --- | --- | --- | --- | --- |
|  |  |  | FF/FFPE dataset 1 | | | | | |  | FF/FFPE dataset 2 | | | | | |  |
|  |  |  | All | | FF | | FFPE | |  | All | | FF | | FFPE | |  |
|  |  | Details | Num. sample | % | Num. sample | % | Num. sample | % |  | Num. sample | % | Num. sample | % | Num. sample | % |  |
|  | Number of samples |  | 31 |  | 19 |  | 12 |  |  | 71 | 100% | 16 | 22.54% | 55 | 77.46% |  |
|  |  |  |  |  |  |  |  |  |  |  |  |  |  |  |  |  |
|  | Sample quality |  |  |  |  |  |  |  |  |  |  |  |  |  |  |  |
|  | Poor quality | AClass Quality Score < 100 | 0 | 0% | 0 | 0% | 0 | 0% |  | 7 | 10% | 0 | 0% | 7 | 13% |  |
|  | Passing sample | AClass Quality Score >= 100 | 31 | 100% | 19 | 100% | 12 | 100% |  | 64 | 86% | 16 | 100% | 48 | 87% |  |
|  |  |  | 31 |  | 19 |  | 12 |  |  | 71 |  | 16 |  | 55 |  |  |
|  |  |  |  |  |  |  |  |  |  |  |  |  |  |  |  |  |
|  | Classification results |  |  |  |  |  |  |  |  |  |  |  |  |  |  |  |
|  | Low conf. AClass | AClass prediction score < 0.7 | 7 | 23% | 3 | 16% | 4 | 33% |  | 9 | 13% | 1 | 6% | 8 | 15% |  |
|  | High conf. AClass | AClass prediction score >= 0.7 | 24 | 77% | 16 | 84% | 8 | 67% |  | 62 | 87% | 15 | 94% | 47 | 85% |  |
|  |  |  | 31 |  | 19 |  | 12 |  |  | 71 |  | 16 |  | 55 |  |  |
|  |  |  |  |  |  |  |  |  |  |  |  |  |  |  |  |  |

Table S7 – Reagent cost breakdown for processing FFPE sample using NanoString and DNA Methylation array platform

|  |  |  |  |  |  |  |  |  |
| --- | --- | --- | --- | --- | --- | --- | --- | --- |
|  | **NanoString** |  |  |  |  |  |  |  |
|  | **Step** | **Item** | **Vendor** | **Number of test supplied** | **Reagent Price ($CAD)** | **Cost/1 sample ($CAD)** |  |  |
|  | Extraction (FFPE) | RNAStorm RNA FFPE Extraction Kit | Cell Data Sciences | 50 | 659.60 | 13.19 |  |  |
|  |  | RNAStorm RNA FFPE Protease | Cell Data Sciences | 50 | 128.25 | 2.57 |  |  |
|  | Nanostring | nCounter Elements Tag Set-36 | NanoString Technologies | 12 | 314.00 | 26.17 |  |  |
|  |  | nCounterMaster Mix | NanoString Technologies | 192 | 7,123.00 | 37.10 |  |  |
|  |  |  |  |  |  |  |  |  |
|  |  |  |  |  |  | **$CAD** | **$USD** |  |
|  |  |  |  |  | **Reagent cost per test:** | **$79.0** | **58.48** |  |
|  |  |  |  |  |  |  |  |  |
|  | **DNA methylation array (Referencing Capper et al., 2018, ActaPatho)** | |  |  |  |  |  |  |
|  | **Step** | **Item** | **Vendor** | **Number of test supplied** | **Reagent Price ($CAD)** | **Cost/1 sample ($CAD)** |  |  |
|  | Extraction (FFPE) | Maxwell 16 FFPE Plus DNA Kit^#^ | Promega | 48 | 613.00 | 12.77 |  |  |
|  | Bisulfide Conversion | Zymo EZ Methylation Kit | Zymo Research | 50 | 226.06 | 4.52 |  |  |
|  | Purification | Zymo DNA Clean Kit | Zymo Research | 25 | 148.65 | 5.95 |  |  |
|  | Restoration | FFPE DNA restoration kit  Infinium HD FFPE DNA Restore Kit | Illumina | 24 | 3,823.96 | 159.33 |  |  |
|  | Methylation array | Inf MethylationEPIC V2.0 Kit | Illumina | 8 | 4,921.48 | 615.19 |  |  |
|  |  |  |  |  |  |  |  |  |
|  | ^#^Original reagent Maxwell 16 FFPE Plus LEV DNA Kit has been discontinued | | |  |  | **$CAD** | **$USD** |  |
|  |  |  |  |  | **Reagent cost per test:** | **$797.8** | **590.34** |  |
|  |  |  |  |  |  |  |  |  |
