# Supplementary material for: Rapid, economical diagnostic classification of ATRT molecular subgroup using NanoString nCounter platform
Source: Neurooncol Adv. 2024 Jan 16;6(1):vdae004. doi: 10.1093/noajnl/vdae004 (PMC10825849; doi:10.1093/noajnl/vdae004)
Supplement: vdae004_suppl_Supplementary_Figures_S1-S6 [file vdae004_suppl_supplementary_figures_s1-s6.pptx]

## Slide 1
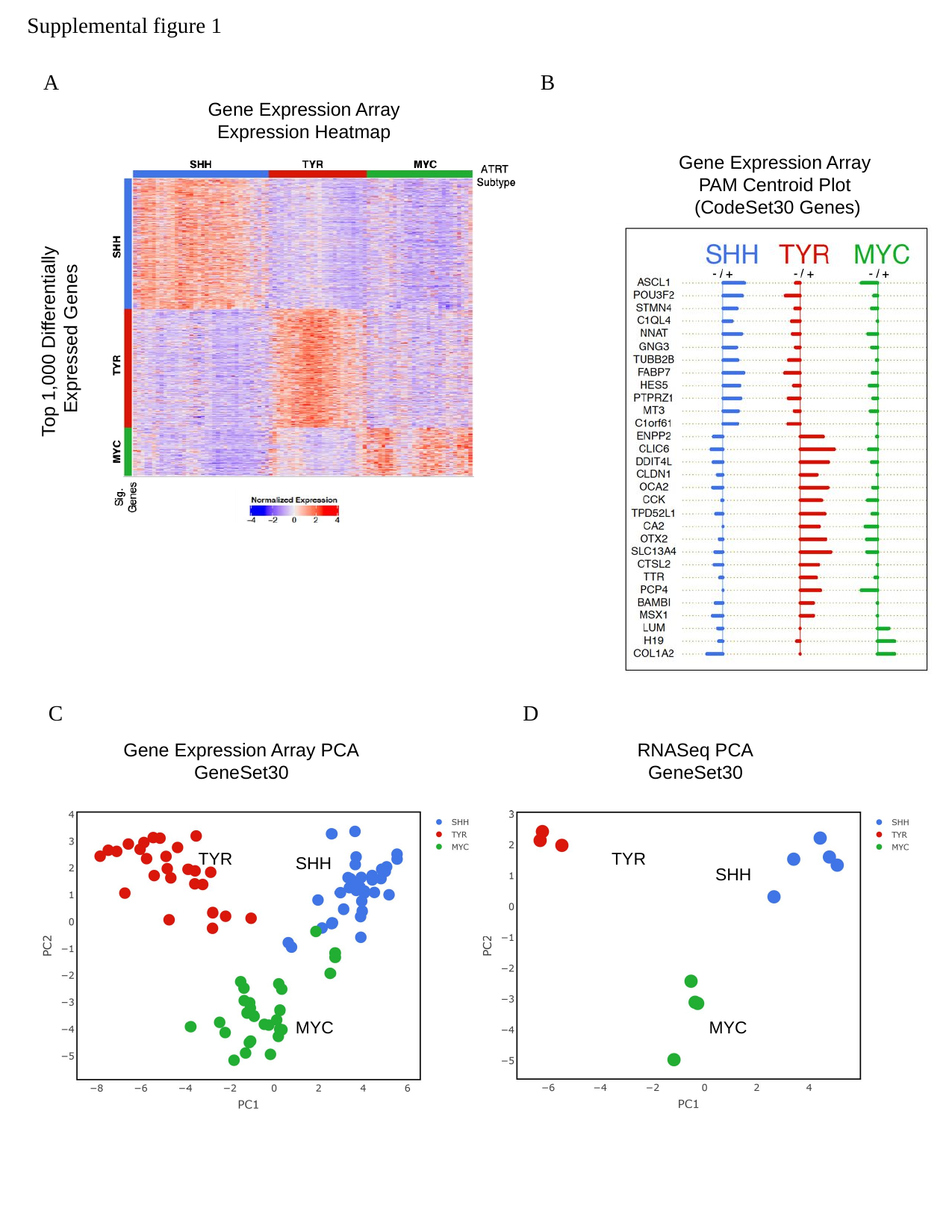

Supplemental figure 1
A
B
Gene Expression Array
Expression Heatmap
Gene Expression Array
PAM Centroid Plot
 (CodeSet30 Genes)
Top 1,000 Differentially
Expressed Genes
C
D
Gene Expression Array PCA
GeneSet30
RNASeq PCA
GeneSet30
TYR
TYR
SHH
SHH
MYC
MYC

## Slide 2
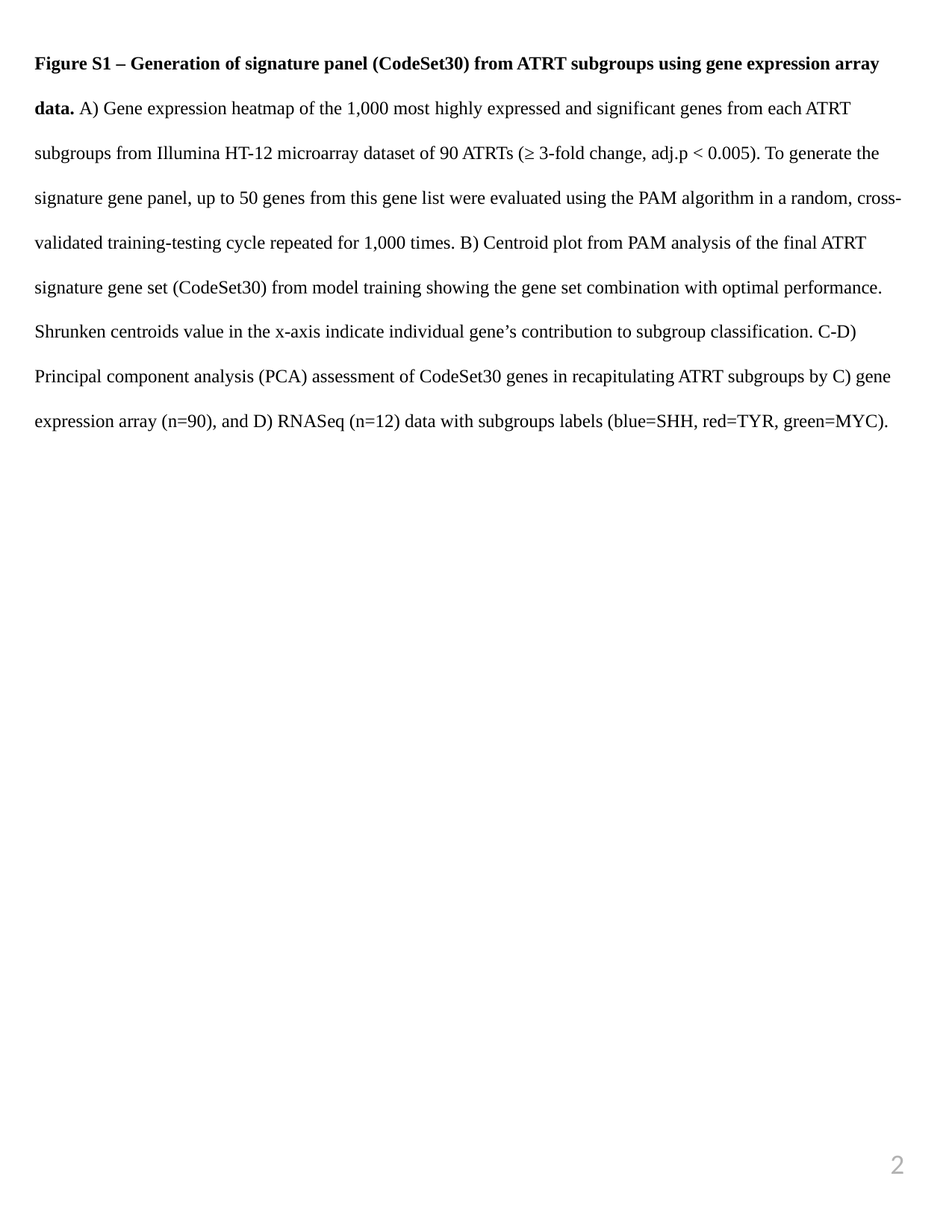

Figure S1 – Generation of signature panel (CodeSet30) from ATRT subgroups using gene expression array data. A) Gene expression heatmap of the 1,000 most highly expressed and significant genes from each ATRT subgroups from Illumina HT-12 microarray dataset of 90 ATRTs (≥ 3-fold change, adj.p < 0.005). To generate the signature gene panel, up to 50 genes from this gene list were evaluated using the PAM algorithm in a random, cross-validated training-testing cycle repeated for 1,000 times. B) Centroid plot from PAM analysis of the final ATRT signature gene set (CodeSet30) from model training showing the gene set combination with optimal performance. Shrunken centroids value in the x-axis indicate individual gene’s contribution to subgroup classification. C-D) Principal component analysis (PCA) assessment of CodeSet30 genes in recapitulating ATRT subgroups by C) gene expression array (n=90), and D) RNASeq (n=12) data with subgroups labels (blue=SHH, red=TYR, green=MYC).
2

## Slide 3
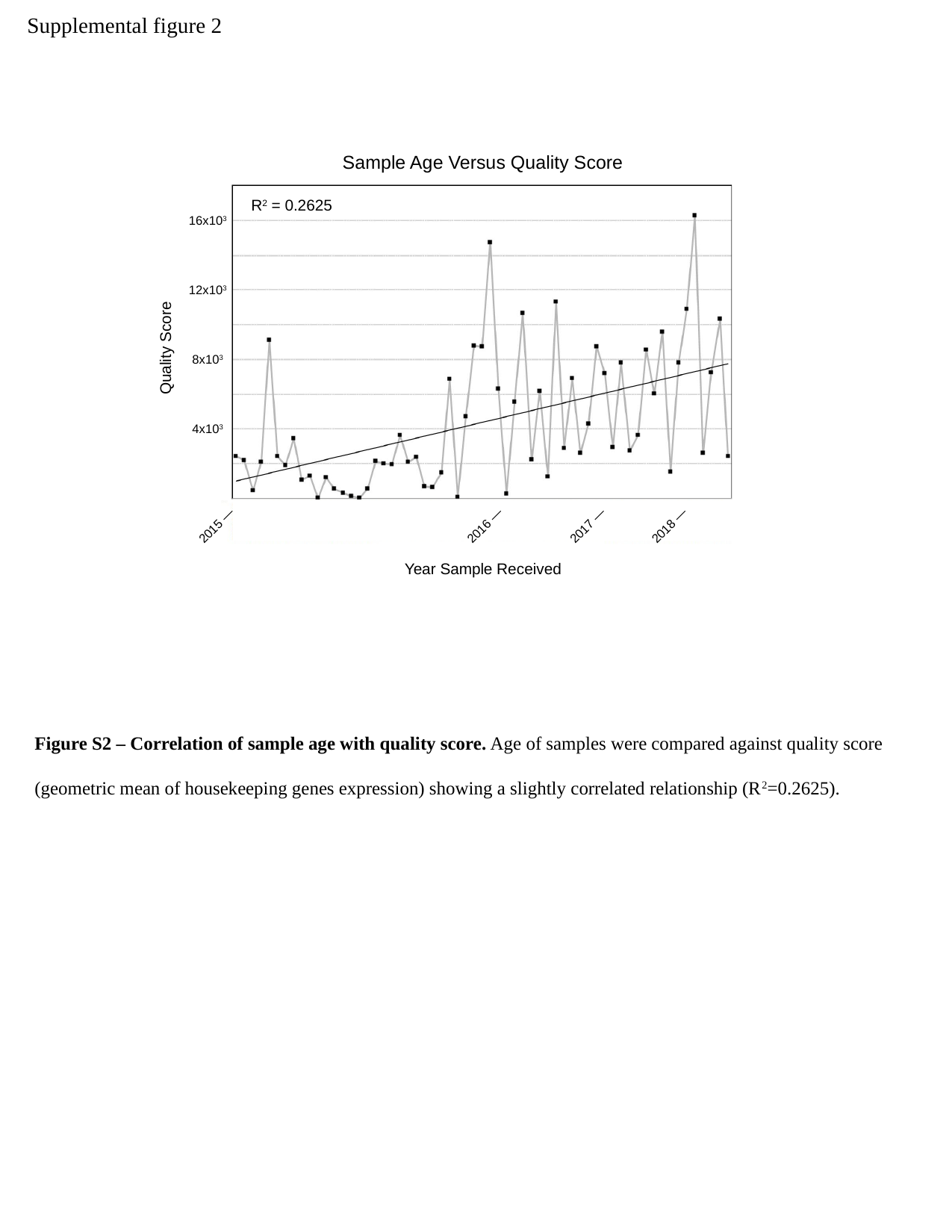

Supplemental figure 2
Sample Age Versus Quality Score
R2 = 0.2625
16x103
12x103
8x10^3^
4x10^3^
Quality Score
2015 —
2016 —
2017 —
2018 —
Year Sample Received
Figure S2 – Correlation of sample age with quality score. Age of samples were compared against quality score (geometric mean of housekeeping genes expression) showing a slightly correlated relationship (R2=0.2625).

## Slide 4
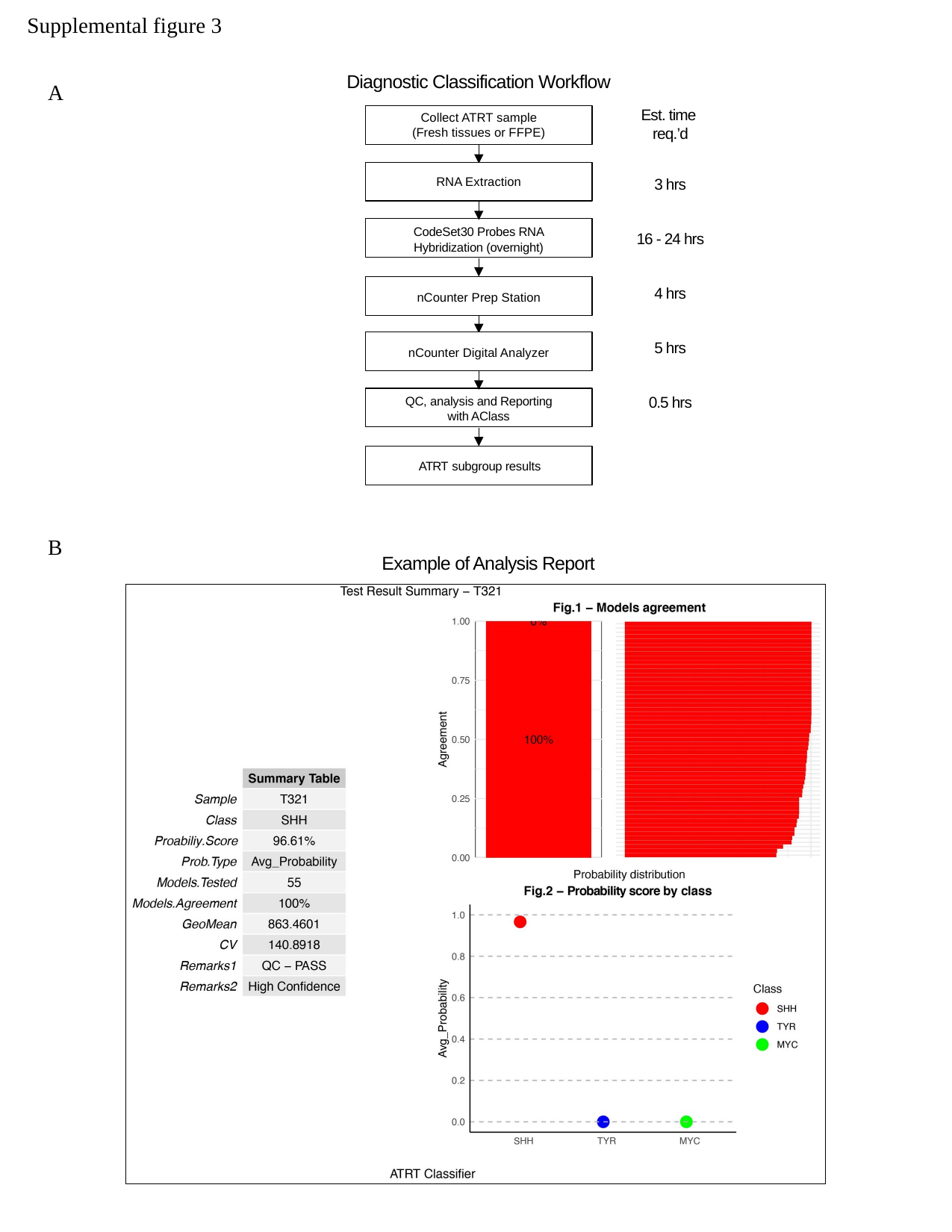

Supplemental figure 3
Diagnostic Classification Workflow
A
Est. time
req.’d
Collect ATRT sample
(Fresh tissues or FFPE)
RNA Extraction
3 hrs
CodeSet30 Probes RNA Hybridization (overnight)
16 - 24 hrs
nCounter Prep Station
4 hrs
nCounter Digital Analyzer
5 hrs
QC, analysis and Reporting with AClass
0.5 hrs
ATRT subgroup results
B
Example of Analysis Report

## Slide 5
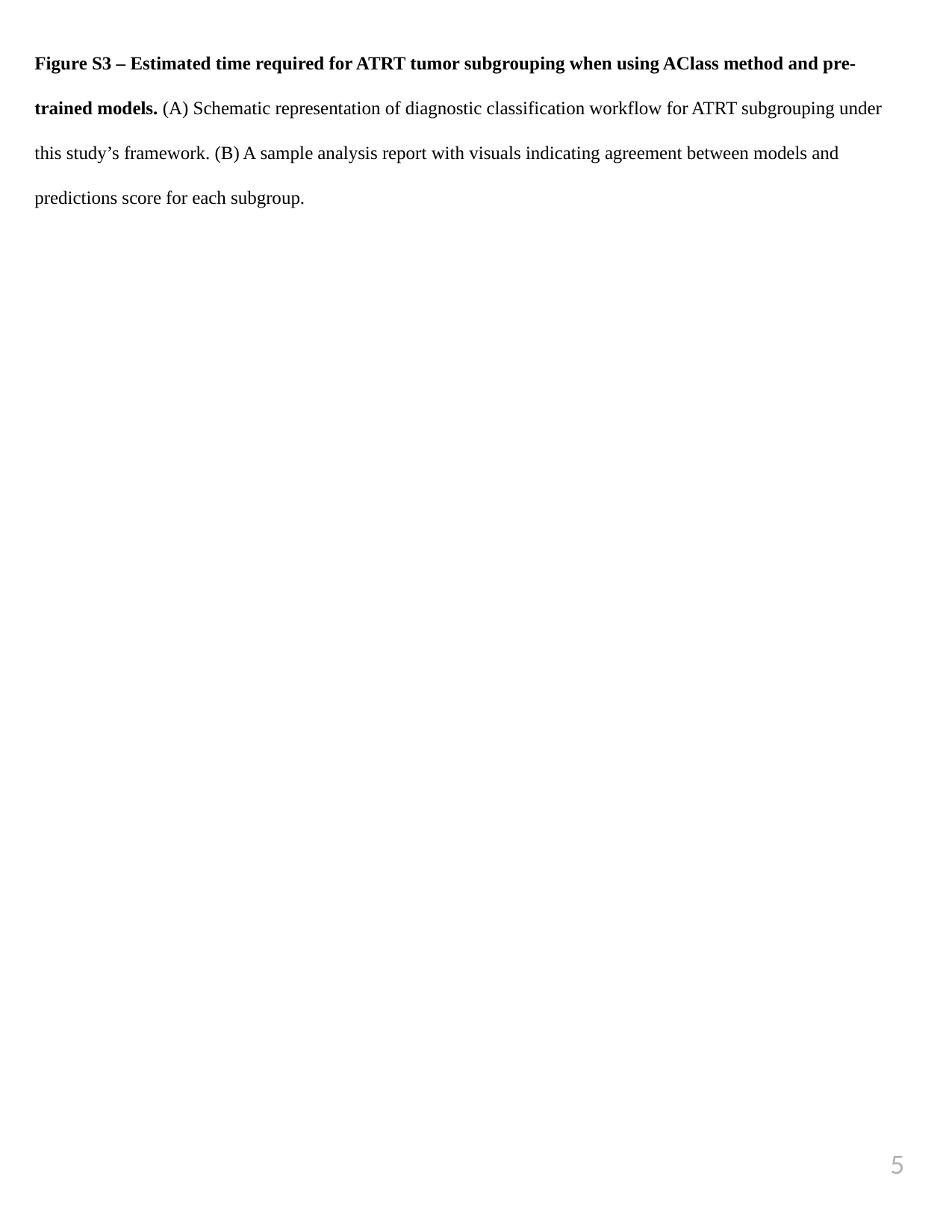

Figure S3 – Estimated time required for ATRT tumor subgrouping when using AClass method and pre-trained models. (A) Schematic representation of diagnostic classification workflow for ATRT subgrouping under this study’s framework. (B) A sample analysis report with visuals indicating agreement between models and predictions score for each subgroup.
5

## Slide 6
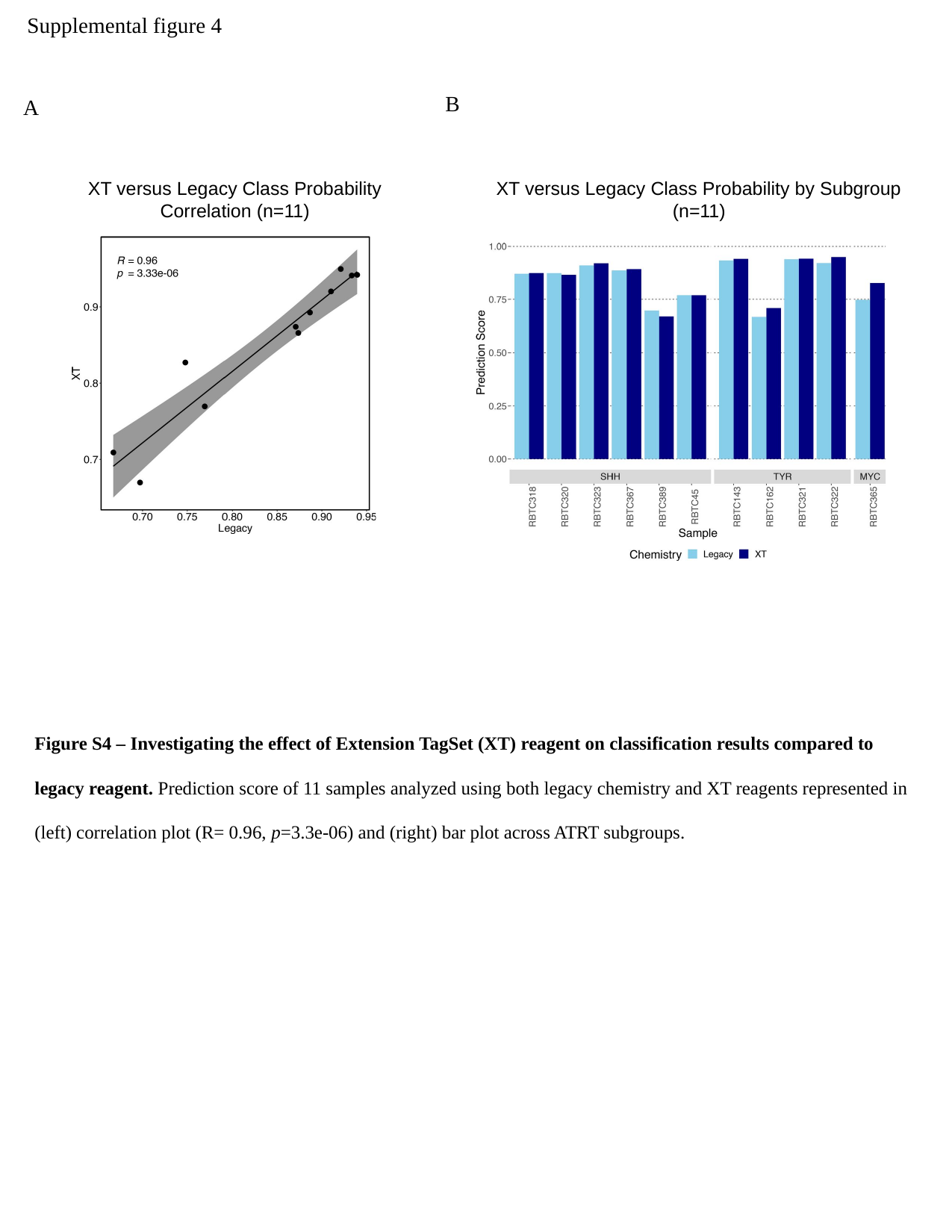

Supplemental figure 4
A
B
XT versus Legacy Class Probability Correlation (n=11)
XT versus Legacy Class Probability by Subgroup
(n=11)
Figure S4 – Investigating the effect of Extension TagSet (XT) reagent on classification results compared to legacy reagent. Prediction score of 11 samples analyzed using both legacy chemistry and XT reagents represented in (left) correlation plot (R= 0.96, p=3.3e-06) and (right) bar plot across ATRT subgroups.

## Slide 7
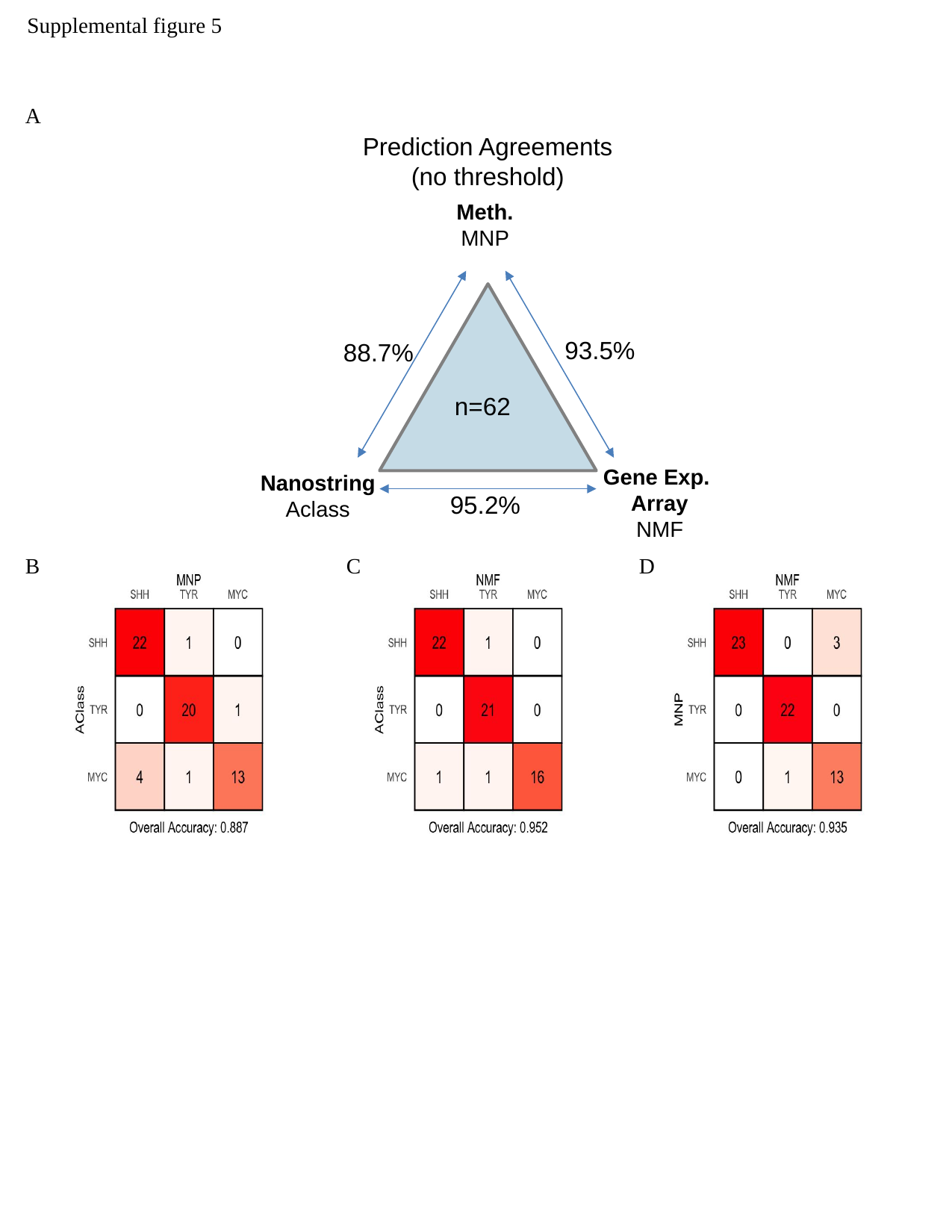

Supplemental figure 5
A
Prediction Agreements
(no threshold)
Meth.
MNP
93.5%
88.7%
n=62
Gene Exp.
Array
NMF
Nanostring
Aclass
95.2%
B
C
D

## Slide 8
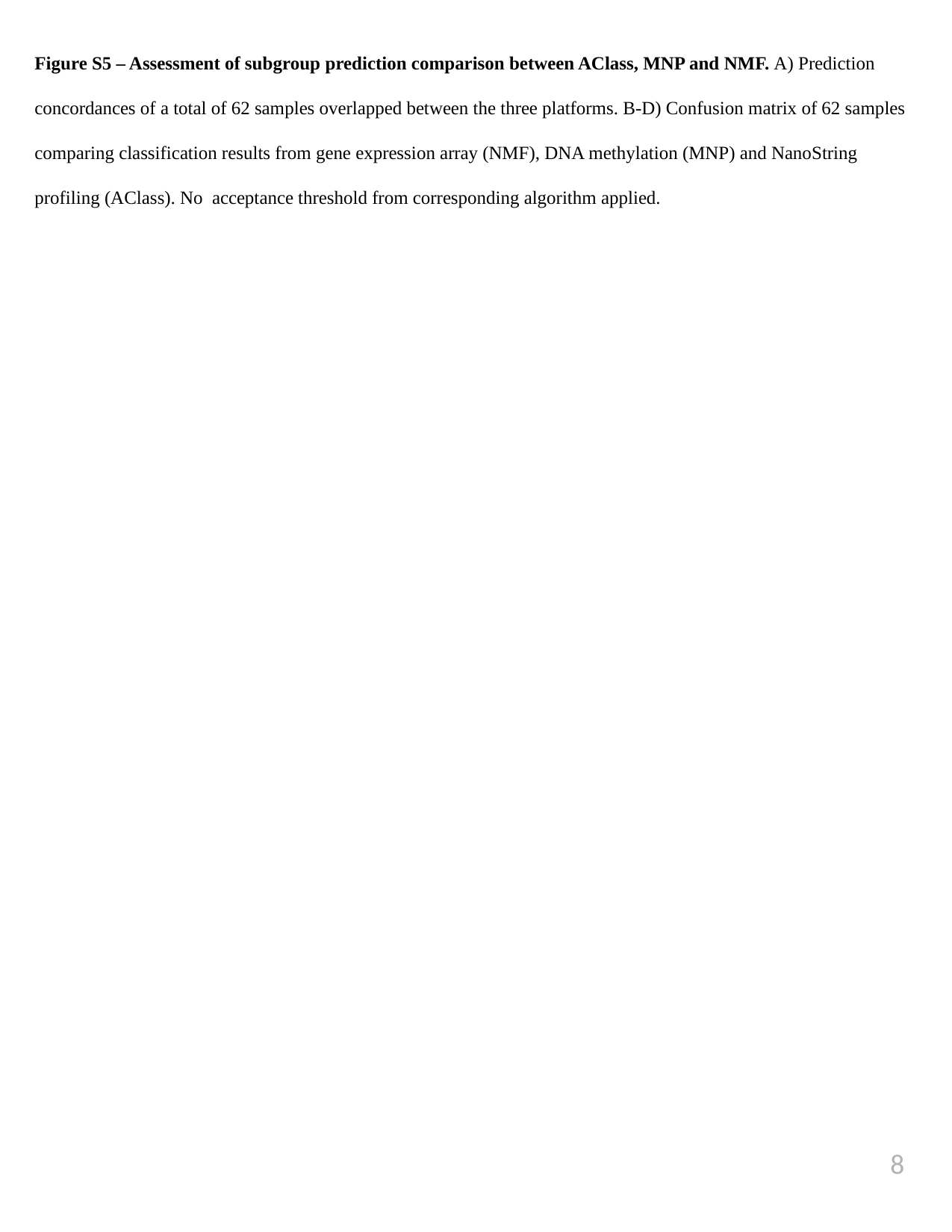

Figure S5 – Assessment of subgroup prediction comparison between AClass, MNP and NMF. A) Prediction concordances of a total of 62 samples overlapped between the three platforms. B-D) Confusion matrix of 62 samples comparing classification results from gene expression array (NMF), DNA methylation (MNP) and NanoString profiling (AClass). No acceptance threshold from corresponding algorithm applied.
8
